# The Genome of the Northern Sea Otter (*Enhydra lutris kenyoni*)

**DOI:** 10.3390/genes8120379

**Published:** 2017-12-11

**Authors:** Samantha J. Jones, Martin Haulena, Gregory A. Taylor, Simon Chan, Steven Bilobram, René L. Warren, S. Austin Hammond, Karen L. Mungall, Caleb Choo, Heather Kirk, Pawan Pandoh, Adrian Ally, Noreen Dhalla, Angela K. Y. Tam, Armelle Troussard, Daniel Paulino, Robin J. N. Coope, Andrew J. Mungall, Richard Moore, Yongjun Zhao, Inanc Birol, Yussanne Ma, Marco Marra, Steven J. M. Jones

**Affiliations:** 1 Canada’s Michael Smith Genome Sciences Centre, British Columbia Cancer Agency, Vancouver, BC V5Z 4E6, Canada; samjones@bcgsc.ca (S.J.J.); gtaylor@bcgsc.ca (G.A.T.); sichan@bcgsc.ca (S.C.); sbilobram@bcgsc.ca (S.B.); rwarren@bcgsc.ca (R.L.W.); shammond@bcgsc.ca (S.A.H.); kmungall@bcgsc.ca (K.L.M.); cchoo@bcgsc.ca (C.C.); hkirk@bcgsc.ca (H.K.); ppandoh@bcgsc.ca (P.P.); aally@bcgsc.ca (A.A.); ndhalla@bcgsc.ca (N.D.); atam@bcgsc.ca (A.K.Y.T.); armellet@bcgsc.ca (A.T.); dpaulino@bcgsc.ca (D.P.); rcoope@bcgsc.ca (R.J.N.C.); amungall@bcgsc.ca (A.J.M.); rmoore@bcgsc.ca (R.M.); yzhao@bcgsc.ca (Y.Z.); ibirol@bcgsc.ca (I.B.); yma@bcgsc.ca (Y.M.); mmarra@bcgsc.ca (M.M.); 2 Department of Medical Genetics, University of British Columbia, Vancouver, BC V6T 1Z3, Canada; 3 Vancouver Aquarium, Vancouver, BC V6G 3E2, Canada; martin.haulena@ocean.org; 4 Department of Molecular Biology and Biochemistry, Simon Fraser University, Burnaby, BC V5A 1S6, Canada

**Keywords:** genome, genome assembly, northern sea otter, *Enhyrda lutris kenyoni*, Mustelidae

## Abstract

The northern sea otter inhabits coastal waters of the northern Pacific Ocean and is the largest member of the Mustelidae family. DNA sequencing methods that utilize microfluidic partitioned and non-partitioned library construction were used to establish the sea otter genome. The final assembly provided 2.426 Gbp of highly contiguous assembled genomic sequences with a scaffold N50 length of over 38 Mbp. We generated transcriptome data derived from a lymphoma to aid in the determination of functional elements. The assembled genome sequence and underlying sequence data are available at the National Center for Biotechnology Information (NCBI) under the BioProject accession number PRJNA388419.

## 1. Introduction

Northern sea otters live in shallow coastal waters in the northern Pacific Ocean. They are the largest member of the Mustelidae family, which also includes weasels, ferrets, and badgers. Unlike most marine animals, sea otters lack blubber and have a very high metabolic rate, eating 20–25% of their body mass in prey, per day [1,2]. In much of their range, their diet consists mainly of herbivorous sea urchins, which in turn preserves the homeostasis of the near-shore ecosystem, consolidating their status as a keystone species [3,4].

Sea otters were hunted to near extinction during the Pacific maritime fur trade. Despite cessation of commercial hunting in 1911 and international protection, the sea otter population has struggled to recover in many areas [5,6]. Protozoan parasites, including *Sarcocystis neurona* and *Toxoplasma gondii*, that may be associated with human activity are a significant source of mortality among southern sea otters, *Enhydra lutris nereis* [7,8,9]. Conservation and recovery efforts improve this by identifying and understanding the threats to sea otter populations. Here, we report the genome sequence of *Enhydra lutris kenyoni*, one of three regional sea otter subspecies. This genome assembly is from the first marine mammal of the Mustelidae family, offering insight into the evolutionary divergence from its terrestrial relatives and providing a platform to study the genetic diversity and population structure of the remaining population.

## 2. Methods, Results, and Discussion

**Figure 1:**
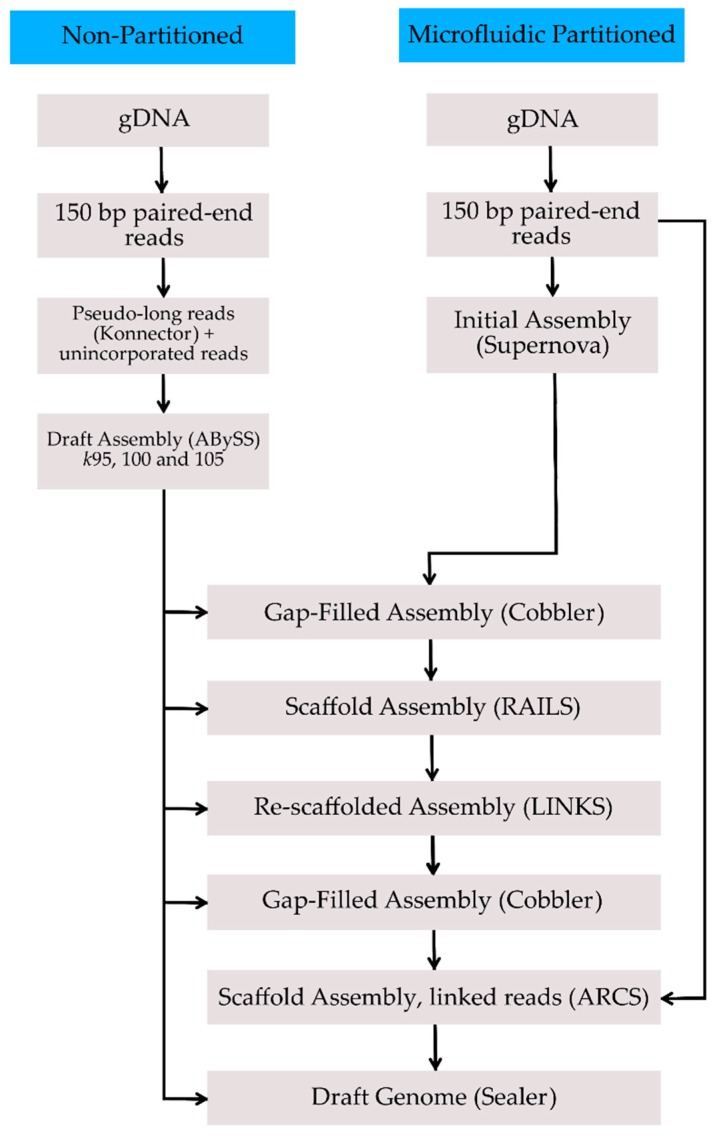
Genome assembly workflow. gDNA: genomic DNA.

The genome was assembled through a combination of paired-end read sequencing libraries and sequence construction using microfluidic partitioning of genomic DNA (gDNA) using the 10x Genomics Chromium System (Figure 1). Sequencing was performed using an Illumina HiSeq X (Illumina, San Diego, CA, USA) instrument at the BC Cancer Agency Genome Sciences Centre (Vancouver, BC, Canada).

The animal under study was a male northern sea otter. “Elfin” was born in 2002 and was rescued as a pup that year in the area of Juneau, Alaska. He subsequently lived at the Vancouver Aquarium. He was diagnosed with lymphoma in 2016, which motivated this work and further studies.

The partitioned library was made as follows: gDNA was extracted from 200 µL of peripheral blood using the QIAGEN MagAttract HMW DNA Kit (QIAGEN, Germantown, MD, USA) and following the HMW gDNA extraction protocol from the Chromium Genome Reagent Kits Version 2 User Guide (PN-120229) (10x Genomics, Pleasanton, CA, USA). The integrity of the gDNA was checked using pulsed-field gel electrophoresis (PFGE). Using the 10x Genomics Chromium Controller instrument fitted with a micro-fluidic Genome Chip (PN-120216) (10x Genomics), a library of Genome Gel Beads was combined with 1 ng of gDNA, Master Mix and partitioning oil to create Gel Bead-In-EMulsions (GEMs). The GEMs were subjected to an isothermic amplification step. Bar-coded DNA fragments were recovered and put through Illumina library construction, following the Chromium Genome Reagent Kits Version 2 User Guide (PN-120229). Quantitative polymerase chain reaction (qPCR) was performed to assess the library yield and an Agilent 2100 Bioanalyzer DNA 1000 chip (Agilent Technologies, Inc., Waldbronn, Germany) was run to determine the library size range and distribution. The library was sequenced on the Illumina HiSeq X sequencer (Illumina), using a paired-end protocol to produce 150 bp reads generating an approximately 60-fold redundant sequence coverage.

A non-partitioned whole-genome PCR-free library was produced as follows: gDNA was extracted from 200 µL of peripheral blood using a QIAamp DNA Blood Mini kit (QIAGEN) on a QIAcube (QIAGEN) and quantified using a Quant-iT double-stranded-DNA high-sensitivity assay (Thermo Fisher Scientific, Waltham, MA, USA). DNA (1000 ng) was acoustically sheared (Covaris Inc., Woburn, MA, USA) to a target size of 300–600 bp. Sheared DNA was end-repaired and PCRClean DX magnetic beads (Aline Biosciences, Woburn, MA, USA) were used to size select ≈450 bp DNA fragments. This was followed by 3′-A-tailing, and then full-length Illumina TruSeq adapters (Illumina) were ligated to the fragments, followed by further magnetic bead purification. A PCR-amplified aliquot of the library was assessed using an Agilent 2100 Bioanalyzer DNA 1000 chip, and a qPCR Library Quantification kit (KAPA, KK4824) (Kapa Biosystems, Wilmington, MA, USA) was used to quantify the PCR-free library concentrations. The genomic library was sequenced in a single lane of a HiSeq X instrument with paired-end 150 base reads, also generating an approximately 60-fold redundant sequence coverage.

**Figure 2:**
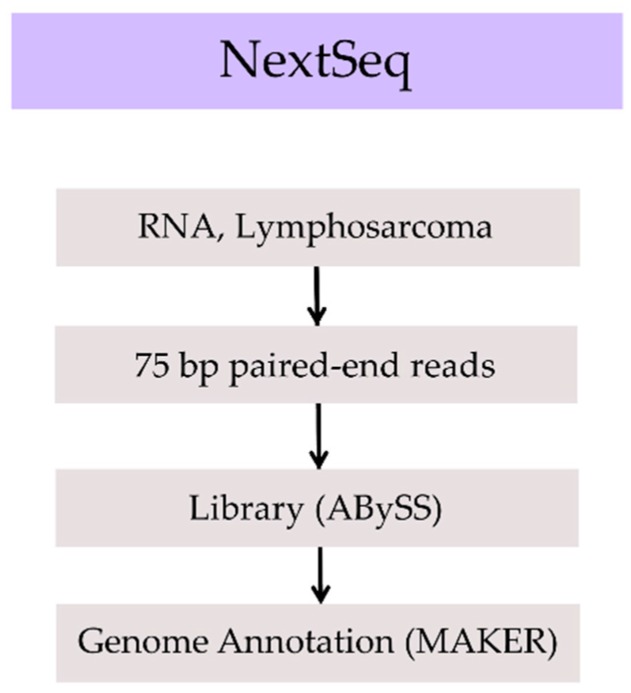
Transcriptome assembly workflow.

Transcript information from RNA sequencing (RNA-Seq) was used to assist genome annotation (Figure 2). The individual under study had been diagnosed with lymphoma, from which RNA was primarily collected to further characterize the disease. We determined that although the RNA would be derived from a pathological specimen, in the absence of other material, it would represent a useful reagent in helping to determine the presence of naturally transcribed regions in the genome. Total nucleic acids were extracted from a lymphosarcoma needle biopsy using the ALINE EvoPure RNA Isolation Kit (Aline Biosciences). Polyadenylated RNA was captured from 400 ng of total RNA using the NEBNext Poly(A) mRNA Magnetic Isolation Module (E7490L, New England Biolabs, Inc., Ipswich, MA, USA) normalized in 35 µL for DNase I-treatment (1 Unit, Invitrogen, Carlsbad, CA, USA). DNase-treated RNA was purified using RNA MagClean DX beads (Aline Biosciences) on a Microlab NIMBUS liquid handler (Hamilton Robotics, Reno, NV, USA). Messenger RNA selection was performed using NEBNext Oligod(T)_25_ beads (New England Biolabs, Inc.), and complementary DNA (cDNA) synthesis and paired-end strand-specific RNA-Seq library construction were performed according to modified protocols [10], in which the random primed cDNA synthesis using a Maxima H Minus First strand cDNA kit (Thermo Fisher, Bartlesville, OK, USA) with actinomycin D and a NEBNext directional second-strand cDNA module (New England Biolabs, Inc.). Libraries were pooled to generate 75 bp paired-end reads on the Illumina NextSeq 500 sequencer (Illumina).

Paired-end sequence reads from the partitioned library were assembled using Supernova (version 1.1.5, 10x Genomics, San Francisco, CA, USA), according to the manufacturer’s instructions. The initial assembly was 2.394 Gbp with a genomic scaffold N50 length of 21.34 Mbp (Table 1). Improvements to the initial assembly were made by incorporating local assemblies using Konnector (version 2.0) [11], which attempts to fill the intervening gap between two paired sequences from a library fragment to provide longer sequences useful for scaffolding. Partitioned and non-partitioned paired-end reads were processed using Konnector to create pseudo-long reads representing completed DNA fragments. All remaining unconnected reads were combined with the pseudo-long reads and assembled by ABySS (version 2.0.1, Canada’s Michael Smith Genome Sciences Centre, Vancouver, BC, Canada), using a range of *kmer* (*k*) values between *k* = 60 and *k* = 120. The assembly using *k* = 100 was the most optimal, generating a scaffold N50 length of 115,235 bp (Table 1).

We followed the same genome scaffolding and finishing methodology employed for the bullfrog genome sequencing project [12]. Briefly, gaps in the supernova draft assembly were initially filled-in using Cobbler (version 0.3, Canada’s Michael Smith Genome Sciences Centre) using the parameters -*d* at 100 and -*i* at 0.95 [13] and utilizing contig sequences from the ABySS assemblies at three *k* values (*k*95, *k*100, and *k*105). A first round of scaffolding was done using RAILS (version 1.2, Canada's Michael Smith Genome Sciences Centre); parameters -*d* at 100 and -*i* at 0.95), an application designed to merge assembly draft sequences and automatically fill-in the resulting gap sequence with support from long DNA sequences, such as contigs from another assembly draft [13]. Here, we used contig sequences from the three aforementioned *kmer* assemblies. The resulting assembly was re-scaffolded three times using LINKS (version 1.8.5, Canada’s Michael Smith Genome Sciences Centre); parameters -*k* at 26; -*l* at 5; -*a* at 0.3; -*d* at 1, 2.5 and 5 kbp; -*t* at 10, and -*o* at 1; incremented at each iteration) [14], using scaffolds from all three ABySS assemblies. Cobbler was used once more to automatically fill remaining gaps with ABySS contig sequences (same parameters). The Assembly Roundup by Chromium Scaffolding application (ARCS) was used to further contiguate the assembly using 10x genomics linked reads (version 1.0.1; ARCS parameters -*s* at 98, -*c* at 5, -*l* at 0, -*d* at 0, -*r* at 0.05, -*e* at 30000, -*v* at 1, -*m* at 20–100,000, and -*z* at 500; LINKS parameters -*l* at 5, and -*a* at 0.09) [15]. A third and final round of gap-filling with Cobbler was applied post-ARCS scaffolding, using the same data and parameters as above. In all, the scaffolding steps yielded an improved scaffold N50 length of 38.45 Mbp and provided the closure of 6298 gaps incorporating 32.2 Mbp of the sequence. Further improvement was achieved with Sealer (version 2.0.2, Canada’s Michael Smith Genome Sciences Centre); parameter -*k* at 90–200, step 10) [16], using sequence contigs from the ABySS *k*100 assembly to further close gaps. In all, Sealer closed an additional 846 gaps.

The final sea otter genome consisted of 2.43 billion bases assembled, with megabase-scale contiguity (N50 length of 38.45 Mbp; Table 1). Within it, Benchmarking Universal Single-Copy Orthologs (BUSCO) [17] identified 5806 highly conserved genes from a potential data set of 6253 genes, of which 93% and a further 4% were present as complete or fragmented protein coding sequences, respectively.

A transcriptome assembly was obtained from the de novo assembly of 75 bp paired-end chastity-passed reads sequenced on the Illumina NextSeq. The library was assembled using ABySS version 1.3.4 and alternate *kmers* from *k*38 to *k*74. MAKER (2.31.9, Yandell Lab, Salt Lake City, UT, USA) was used to provide an automated gene annotation and the determination of the protein coding potential of the genome assembly [18]. Low-complexity sequences were masked to reduce the size of the genome being annotated using the RepeatMasker [19] program embedded in MAKER. Following repeat masking, three *ab initio* gene predictors, AUGUSTUS [20], SNAP [21], and GeneMark [22], were used to combine the results into a single gene set. AUGUSTUS predictions were based on the *Homo sapiens* training set; SNAP was trained using the CEGMA predicted genes (CEGMA, version 2.5) [17]; GeneMark is self-trained. RNA-Seq data was used to inform the gene prediction tools. MAKER predicted 33,012 transcripts from 28,228 genes with experimental support. The average predicted protein length was 471 amino acids. An official Refseq annotation of this genome was also generated for this genome assembly and is available from the Refseq website (https://www.ncbi.nlm.nih.gov/refseq/).

The sea otter genome assembly compared favorably with the previous most contiguous *Mustela* genome, *putorius furo* (ferret) [23], with a reconstructed genome size of 2.41 Gbp and scaffold N50 value of 9.3 Mbp. In terms of assembly completeness as reported by BUSCO, a complete copy of 92.85% of BUSCO genes was identified in the sea otter assembly, whereas 92.30% were detected in the ferret genome. While separated by approximately 17.5 million years of evolutionary time (timetree.org), a *kmer* Bloom filter-based genome similarity analysis (performed as described in [24]) indicated that the sea otter and ferret genomes were approximately 95.65% ± 1.45 × 10^5^% (mean ± standard deviation) identical at the nucleotide level. Our approach exemplifies the utility of microfluidic partition libraries to produce highly contiguous mammalian-size reference-quality assemblies. Furthermore, we demonstrate how assemblies can be improved with the incorporation of assembled non-partitioned genomic libraries. We note that incorporating data from independent non-partitioned libraries provides only a modest overall improvement in the assembly, indicating that the partitioned libraries show no obvious bias. In the future, incorporating an independent assembly on the partitioned data may be an equally useful approach to improve assembly contiguity and eliminate the need for a second library.

## Figures and Tables

**Table 1:** Assembly statistics and gene content for the genome sequences reported in this study.

Assembly	Total Size (Gbp)	No. of Gaps	No. of Scaffolds	Scaffold N50 (Mbp)	Longest Scaffold (Mbp)	BUSCO Complete Genes	BUSCO Complete + Fragmented Genes
ABySS-pe	2.555 + 0.132% in gaps	121,917	70,247	0.115	2.301	5534 (88.50%)	5931 (94.85%)
Supernova	2.394 + 1.27% in gaps	26,442	10,285	21.34	97.14	5785 (92.52%)	6047 (96.71%)
Rails/Cobbler	2.426 + 1.23% in gaps	23,659	6770	38.45	107.2	5806 (92.85%)	6051 (96.77%)
Sealer	2.426 + 1.22% in gaps	22,813	6770	38.45	107.2	5806 (92.85%)	6051 (96.77%)

BUSCO: Benchmarking Universal Single-Copy Orthologs.
